# Supplementary material for: Competitive binding of actin and SH3 domains at proline-rich regions of Las17/WASP regulates actin polymerisation
Source: Commun Biol. 2025 May 15;8:759. doi: 10.1038/s42003-025-08188-4 (PMC12081870; doi:10.1038/s42003-025-08188-4)
Supplement: Supplementary file 2 — Supplementary Information [file 42003_2025_8188_MOESM2_ESM.pdf]

Supplementary Information.

Methods used in Supplementary Data

Yeast strains, growth and staining

Yeast plasmids used in this study are listed in Supplementary table 1 and yeast strains used in Supplementary table 2. Unless stated otherwise, cells were grown with rotary shaking at 30°C in liquid YPD medium (1% yeast extract, 2% Bacto-peptone, 2% glucose supplemented with 40 µg/ml adenine) or in synthetic medium (0.67% yeast nitrogen base, 2% glucose) with appropriate supplements. Growth assays used solid medium with 2% agar added to above. All strains carrying fluorescent tags have growth properties similar to isogenic control strains with the exception of strains carrying dual reporters where lifetimes were longer for both wild type and mutant strains (in Figure 9D). Point mutations in *LAS17* were generated using site directed mutagenesis (Agilent). DNA cassettes carrying mutations for integration were transformed into KAY1801. Mutant colonies were counter-selected on minimal medium, containing 0.005% uracil and 0.1% 5'-Fluoroorotic Acid (5-FOA; Melford labs). Allele exchange, in growing $Ura3^-$ 5-FOA resistant colonies, was confirmed by PCR and sequencing. Halo assays were performed as described [1].

Rhodamine- phalloidin was used to fix and stain yeast cells to visualise F-actin structures as described[2]. In brief, an overnight culture was refreshed by adding 0.5 ml of the culture into 4.5 ml of fresh media and cells were then grown for 4 hrs at 30°C. 1 ml of actively growing yeast cells (OD_{600} 0.1-0.3 U) were fixed by addition of 134 µl of 37% formaldehyde for 1 hr at room temperature. Cells were spun down for 3 min at 3000 *g* followed by two washes with 500 µl of wash buffer 1 (1x phosphate buffered saline (PBS) pH 7.4, 1 mg/ml BSA and 0.1% Triton X-100). The pellet was resuspended in 50 µl of wash buffer 1 with 5 µl rhodamine phalloidin and samples were incubated in the dark for 30 mins. The cells were spun down and washed twice with 50 µl of wash buffer 2 (1x PBS and 1 mg/ml BSA). The pellet was resuspended in 200 µl of wash buffer 2 and then 2 µl of each sample was spotted onto a microscope slide. Cells were imaged as described below.

Lucifer yellow endocytic uptake assays are as described [3] using 40 mg/ml lucifer yellow (LY-CH dilithium salt), incubation at 30°C for 90 minutes. Cells were washed three times with 1 ml of ice-cold succinate/azide buffer (50 mM succinic acid, 20 mM sodium azide buffer (pH5) before imaging.

Supplementary Table 1: Plasmids used in this study

pKA	Description	Origin/Reference
566	GST-Las17 (300-422)	Ref
671	GST-Las17 (300-633)	Ref
1284	GST-Las17 (342-394) minimal component	This study
928	Sla1-SH3#1 (3-68) in pGEX4T1	[4]
1247	Sla1-SH3#1-2 (5-131) in pGEX6P1	This study
1248	Sla1-SH3#1-3 (5-413) in pGEX6P1	This study

1280	His-Las17 (300-422) pTOPO 6xHis-Las17 (300-422) as a BamH1-HindIII fragment	This study
748	GST-Ysc4-SH3 in pGEX4T1	[4]
947	GST-Sla1 SH3#2 in pGEX4T1	[4]
927	Sla1-SH3#3 (354-413) in pGEX4T1	[4]
1317	Sla1-SH3#1-2 (5-131) W108A	This study
1189	GST-Las17 (300-422) RR(349,350)AA	Ayscough lab
1190	GST-Las17 (300-422) RR(382,383)AA	Ayscough lab
1191	GST-Las17 (300-422) RR(349,350)AA and RR(382,383)AA	[5]
1336	GST-Las17 (300-422) RR(319,322)AA	This study
1337	GST-Las17 (300-422) RR(319,322)AA, RR(349,350)AA, and RR(382,383)AA	This study
1252	GST-Sec4 (full length) cloned in BamH1 and Sal1 sites of pGEX-6P1	This study
1278	GST-Las17 (300-422) P387A	This study
1279	GST-Las17 (300-422) P388A	This study
417	pGEX6P-1 (GST alone)	Pharmacia/GE Healthcare
88	GFP-ABP1, URA, CEN	[6]

Supplementary Table 2: Yeast strains

Strain	Genotype	Origin
KAY389	*MAT***a** *his3*-Δ*200 leu2*-3,112 *ura3*-52 *trp1-1 lys2-801*	KA Lab
KAY1801	*MAT***a** *his3*-Δ*200 leu2*-3,112 *ura3*-52 *trp1-1 lys2-801 las17*Δ*::URA3*	[7]
KAY1912	*MAT***a** *his3*-Δ*200 leu2*-3,112 *ura3*-52 *trp1-1 lys2-801 LAS17-7xAla-GFP::kanMX6*	[7]
KAY1937	*MAT***a** *his3*-Δ*200 leu2*-3,112 *ura3*-52 *trp1-1 lys2-801 las17*P387A*::URA3*	This study
KAY1938	*MAT***a** *his3*-Δ*200 leu2*-3,112 *ura3*-52 *trp1-1 lys2-801 las17*P387A-*7Ala-GFP::KanMx*	This study
KAY1947	*MAT***a** *his3*-Δ*200 leu2*-3,112 *ura3*-52 *trp1-1 lys2-80, ARC15-mCherry::HIS3*	[7]
KAY1948	*MAT***a** *his3*-Δ*200 leu2*-3,112 *ura3*-52 *trp1-1 lys2-801 las17P387A::URA3, ARC15-mCherry::HIS3*	This study
KAY1949	*MAT***a** *his3*-Δ*200 leu2*-3,112 *ura3*-52 *trp1-1 lys2-801, LAS17-7xAla-GFP::kanMX6, ARC15-mCherry::HIS3*	[7]
KAY1950	*MAT***a** *his3*-Δ*200 leu2*-3,112 *ura3*-52 *trp1-1 lys2-801 las17*P387A-*7Ala-GFP::KanMx, ARC15-mCherry::HIS3*	This study

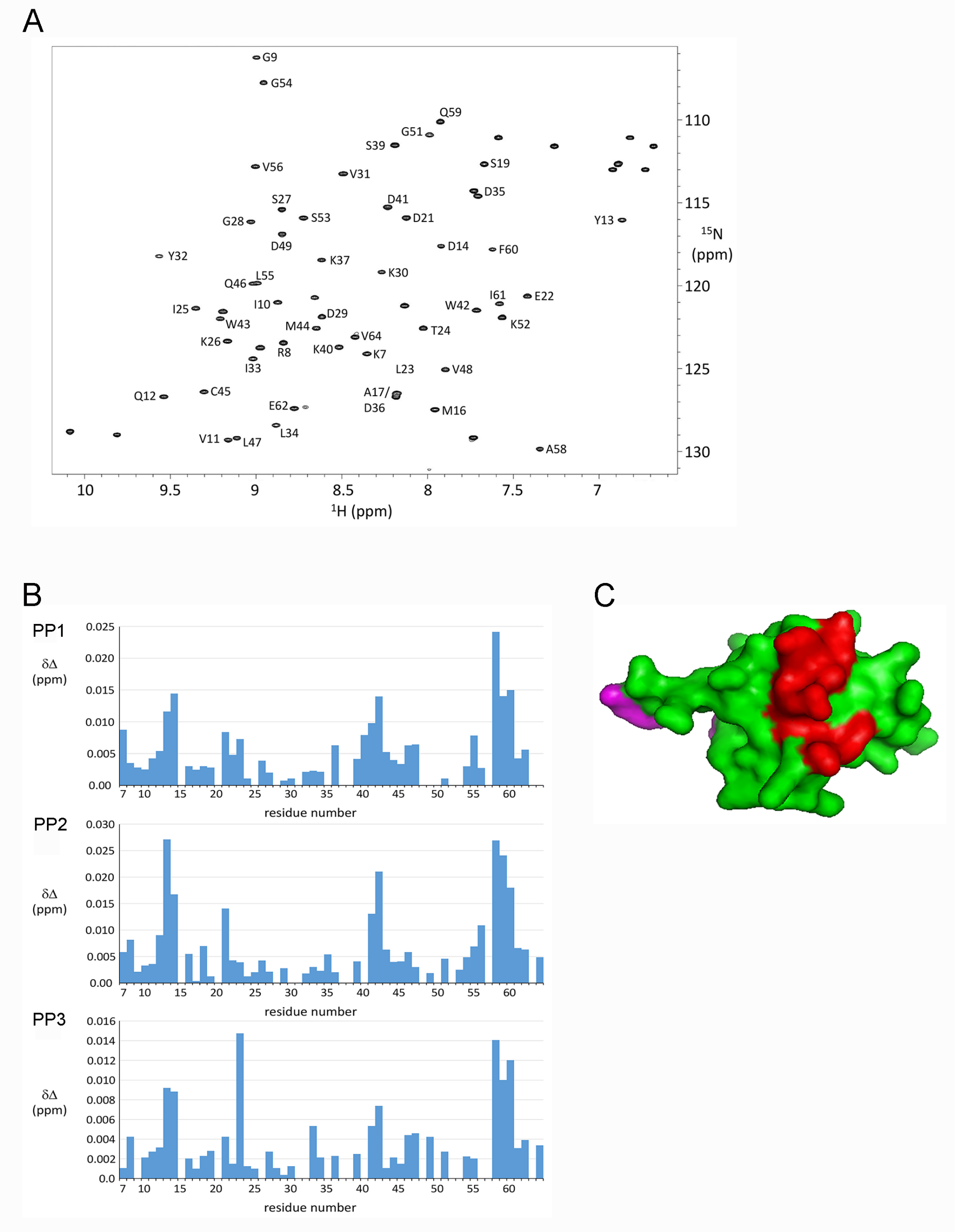

Supplementary Figure 1. NMR titrations of Sla1 SH3 domain 3 with peptides

(A) Assignment of the ^{1}H-^{15}N HSQC spectrum of Sla1 SH3 domain 3. Unlabelled signals are either sidechain or from the largely unstructured N- and C-termini. (B) Chemical shift changes for Sla1 SH3#3 on addition of a 5-fold excess of the peptides PP1, PP2 and PP3 based on the three polyproline/ actin binding sites. Chemical shifts are shown as weighted chemical shift changes for ^{1}H and ^{15}N, $[\Delta\delta_H^2 + (0.14\Delta\delta_N^2)]^{1/2}$. (C) The peptide binding site on Sla1 SH3 domain 3. Chemical shift changes on addition of peptides were used to calculate the mean and standard deviation weighted shift change for each protein residue. Residues with shift changes larger than (mean + sd) are indicated in red on the figure, and comprise Y13, D14, D41, W42, A58, Q59 and F60. The residue in magenta indicates the C terminus.

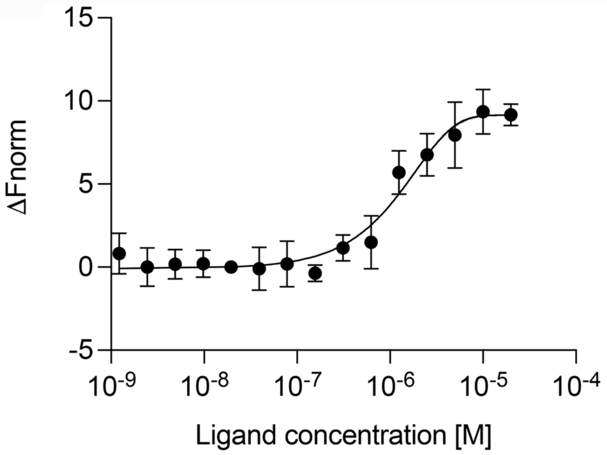

Supplementary Figure 2. Measuring affinity of Las17(342-392) binding to actin. Microscale thermophoresis assay trace to measure affinity of Las17(342-392) binding to actin. Las17(342-392) was added to labelled actin as described at concentrations from 1.2 nM – 20 µM. Error is standard error of the mean.

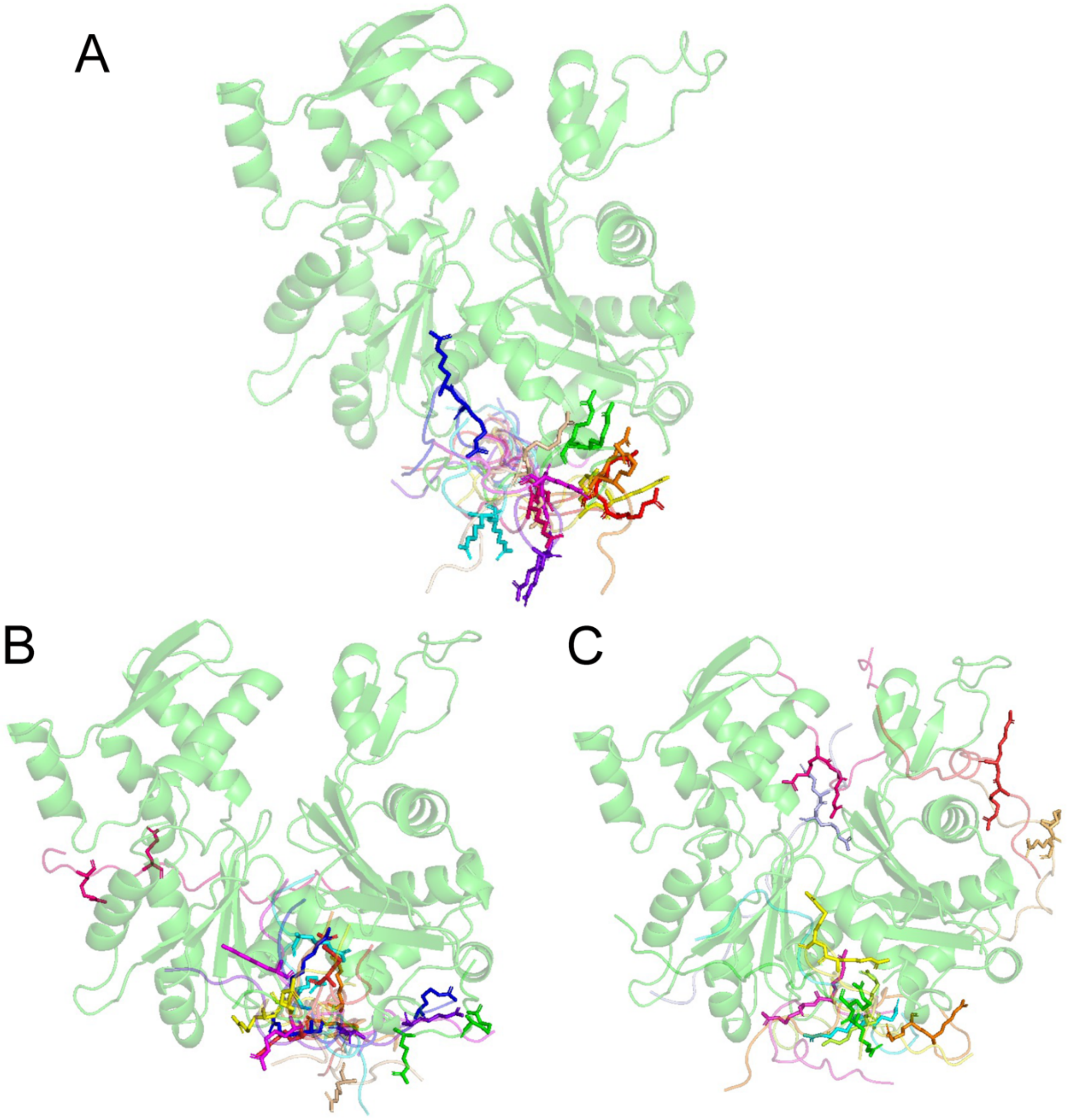

Supplementary Figure 3. Modelling the Las17-Actin interactions. All residues are shown as a cartoon ribbon with the exception of arginines flanking the polyproline sequence of the ABS sites and key interacting actin residues. Yeast G-actin is shown in green (PDB: 1YAG). The HPEPDOCK program was used to model both ABS1 (A) and ABS2 (B) peptide binding to actin. These both showed a predominance of binding of the peptides to the barbed end groove, though the orientation of the peptide was in either of two orientations with interactions of flanking arginines at each end of the peptide with acidic residues in actin. (C) FLEXPEPDOCK was used to investigate peptide binding of ABS3. The top ten predicted models (shown – each in different colour) indicate binding in the barbed end groove of actin.

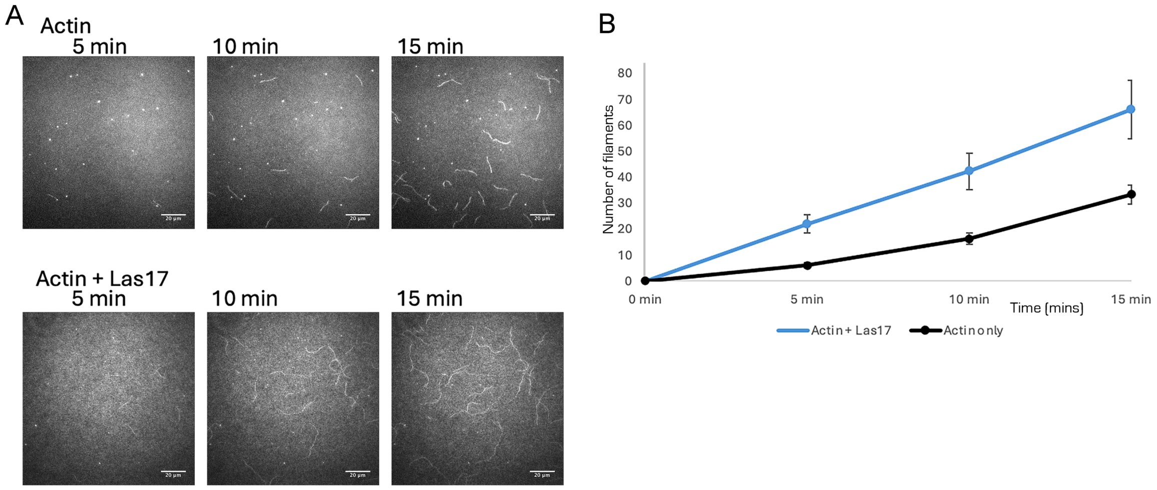

Supplementary Figure 4. Impact of Las17 on actin filament number. Actin filaments were polymerised in the presence or absence of Las17. Filament formation was followed using TIRF as described in [8]. (A) Stills from movies also provided in Supplementary materials. (B) The number of filaments was measured on image stills at 5, 10 and 15 minutes from 3 independent experimental repeats. Each image still was 136 μm^2. Errors bars are standard deviation.

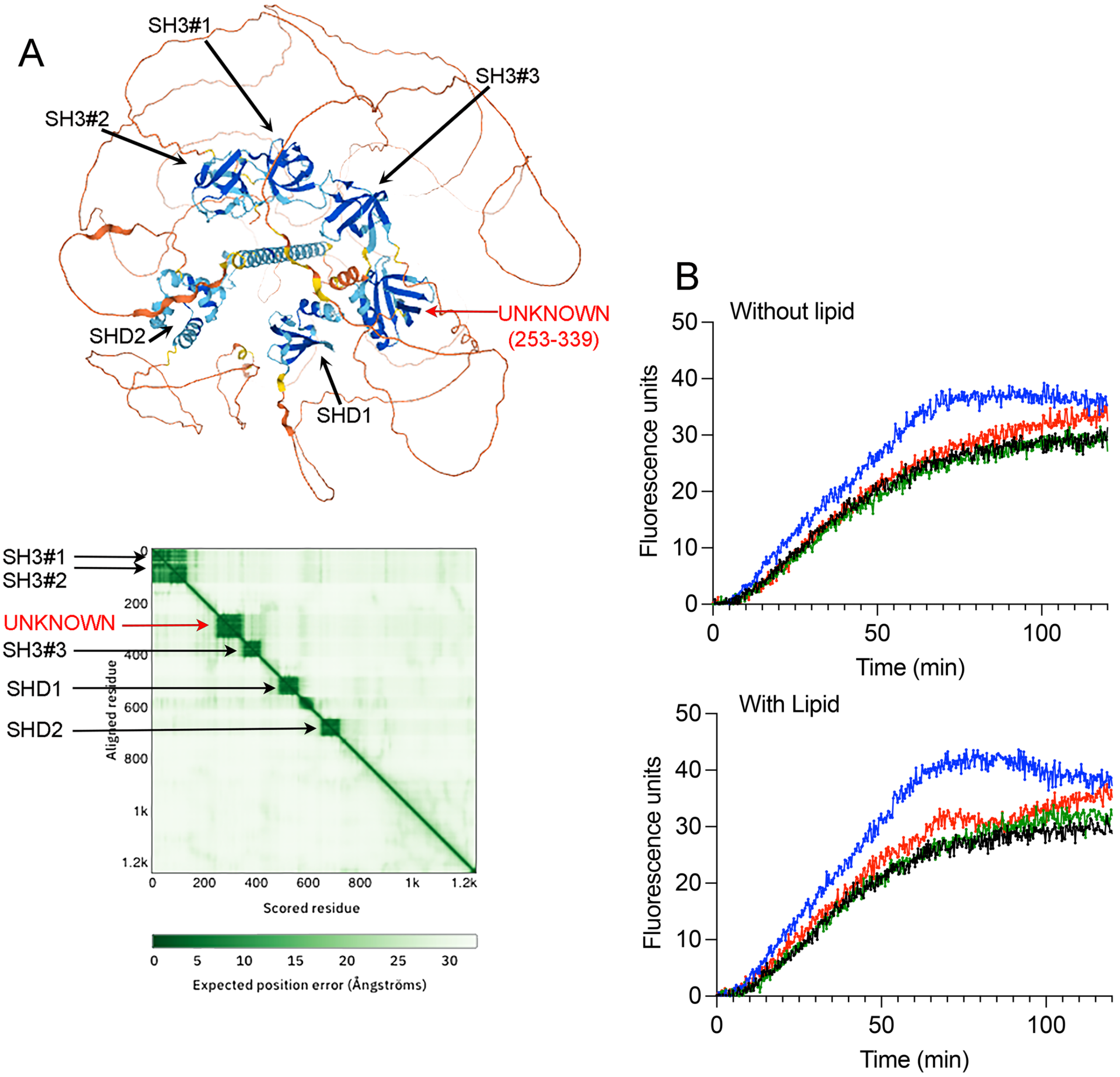

Supplementary Figure 5. Identification of unknown domain in Sla1. (A) Alphafold domain schematic (upper panel) and domain probability (lower panel). Arrows indicate known (black) and the unknown (red) domains. The domain was subsequently shown to be a lipid binding PH domain, figure 7. (B) Pyrene actin assay showing Sla1 inhibition of Las17 fragment (300-633) activity in the absence (upper panel) and presence (lower panel) of 5.5mg/ml Folch fraction liposomes. Actin only (black), actin and Las17 (blue), actin and Sla1 (green), actin, Las17 and Sla1 (red). Actin; 3 μM, Las17; 300nM, Sla1; 300nM. As shown addition of liposomes to the assay did not relieve the inhibition of Sla1 on Las17-mediated actin polymerisation.

Supplementary Figure 6. *Las17 P387A* rescues the temperature sensitivity of *las17Δ* but alters actin dynamics, organisation and endocytosis. (A) Serial dilution of *S. cerevisiae* shows temperature sensitivity of *las17Δ* but not of *las17 P387A*. (B) Growth of *Las17* and *las17 P387A* expressing strains on plates on which a disc carrying 10 µl of latrunculin-A of varying concentrations was placed (white disc). Yeast growth is prevented (dark zone) by concentrations of latrunculin-A that are toxic to the cells. The calculated increase in Lat-A sensitivity for Las17 P387A was 7-fold compared to wild type based on 5 repeats. (C) Rhodamine-phalloidin was used to stain F-actin in cells. Representative images from each strain are shown. >100 cells were visualised and their actin organisation categorised as indicated by key. Size bar = 5 µm. Errors are SEM from 3 independent experiments. (D) Cells were incubated with the fluid phase dye Lucifer yellow for 90 minutes. They were then scored according to the extent of uptake and categorised according to the key shown. >100 cells were visualised for each strain in each of 3 independent experiments. Size bar = 2 µm.

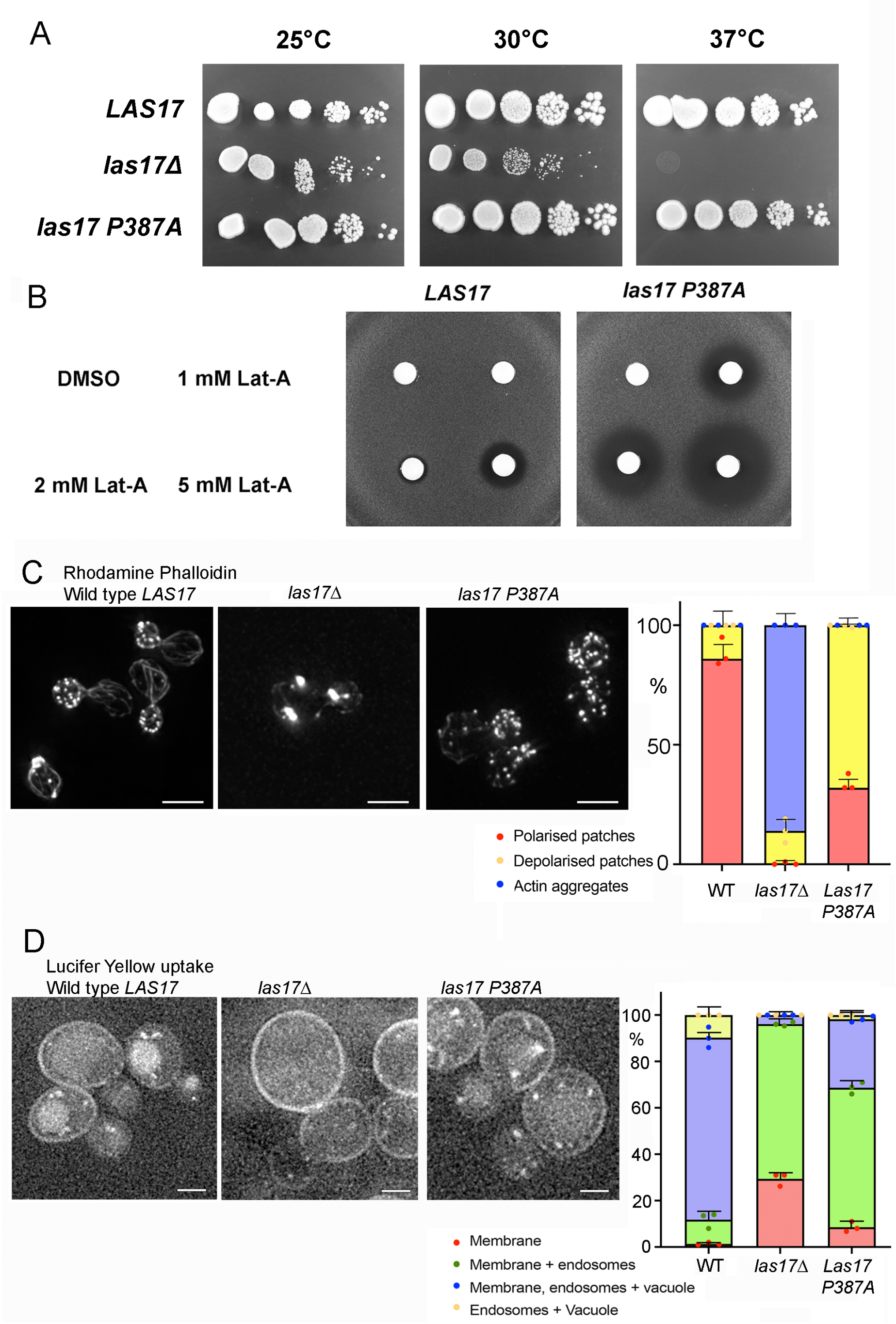

Supplementary Figure 7. Full raw gel image with standards for Figure 7

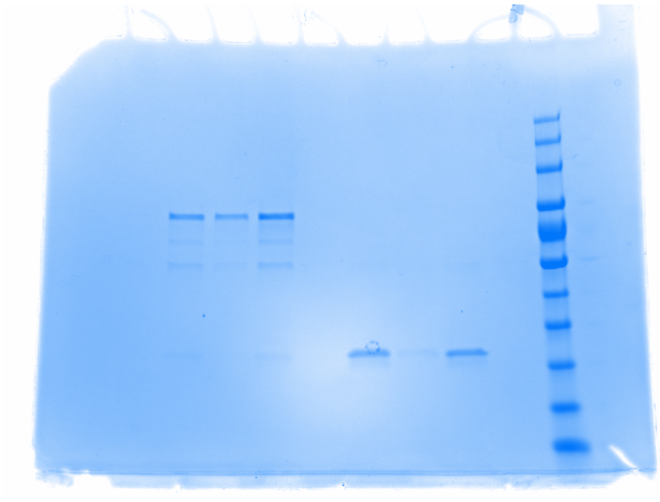

Supplementary references

1 Ayscough, K. R. *et al.* High rates of actin filament turnover in budding yeast and roles for actin in establishment and maintenance of cell polarity revealed using the actin inhibitor latrunculin-A. *J Cell Biol* **137**, 399-416, doi:10.1083/jcb.137.2.399 (1997).

2 Pringle, J. R. *et al.* Fluorescence microscopy methods for yeast. *Methods Cell Biol* **31**, 357-435, doi:10.1016/s0091-679x(08)61620-9 (1989).

3 Dulic, V. *et al.* Yeast endocytosis assays. *Methods Enzymol* **194**, 697-710, doi:10.1016/0076-6879(91)94051-d (1991).

4 Tong, A. H. *et al.* A combined experimental and computational strategy to define protein interaction networks for peptide recognition modules. *Science* **295**, 321-324, doi:10.1126/science.1064987 (2002).

5 Allwood, E. G., Tyler, J. J., Urbanek, A. N., Smaczynska-de, R., II & Ayscough, K. R. Elucidating Key Motifs Required for Arp2/3-Dependent and Independent Actin Nucleation by Las17/WASP. *PLoS One* **11**, e0163177, doi:10.1371/journal.pone.0163177 (2016).

6 Doyle, T. & Botstein, D. Movement of yeast cortical actin cytoskeleton visualized in vivo. *Proc Natl Acad Sci U S A* **93**, 3886-3891, doi:10.1073/pnas.93.9.3886 (1996).

7 Tyler, J. J. *et al.* Phosphorylation of the WH2 domain in yeast Las17/WASP regulates G-actin binding and protein function during endocytosis. *Sci Rep* **11**, 9718, doi:10.1038/s41598-021-88826-z (2021).

8 Graziano, B. *et al.* -Mechanism and cellular function of Bud6 as an actin nucleation-promoting factor. *Mol Biol Cell.* **22**, 4016-4028 (2011).
