# Supplementary material for: Competitive binding of actin and SH3 domains at proline-rich regions of Las17/WASP regulates actin polymerisation
Source: Commun Biol. 2025 May 15;8:759. doi: 10.1038/s42003-025-08188-4 (PMC12081870; doi:10.1038/s42003-025-08188-4)
Supplement: Supplementary file 3 — Description of Additional Supplementary Files [file 42003_2025_8188_MOESM3_ESM.pdf]

Description of Additional Supplementary Files

File name: Supplementary Data 1

Description: Numeric Data for Fig 1

File name: Supplementary Data 2

Description: Text file data for Data for Fig 2b

File name: Supplementary Data 3

Description: Numeric data for Fig 2c, 3d

File name: Supplementary Data 4

Description: Text file data for Data for Fig3c

File name: Supplementary Data 5

Description: Numeric data for Fig 4,6,7,8,9

File name: Supplementary Data 6

Description: CelluSpots peptide array identities for SPOT assay in Figure 8

File name: Supplementary Video 1

Description: Actin polymerising alone

File name: Supplementary Video 2

Description: Actin polymerising in the presence of las17

File name: Supplementary Video 3

Description: Actin polymerising in the presence of las17 and Arp2/3

File name: Supplementary Video 4

Description: All three videos in a single format so they can be viewed alongside
